# Social determinants of health associated with hemodialysis non-adherence and emergency department utilization: a pilot observational study

**DOI:** 10.1186/s12882-019-1673-7

**Published:** 2020-01-06

**Authors:** Kamna S. Balhara, Lori Fisher, Naya El Hage, Rosemarie G. Ramos, Bernard G. Jaar

**Affiliations:** 1 The Johns Hopkins University School of Medicine, Baltimore, MD USA; 2 University of the West Indies, Mona, Jamaica; 3 West Penn Hospital, Pittsburgh, PA USA; 4 The University of Texas Health Science Center at San Antonio, San Antonio, TX USA; 5 The Johns Hopkins Bloomberg School of Public Health, Baltimore, MD USA; 6 The Welch Center for Prevention, Epidemiology, and Clinical Research, Baltimore, MD USA; 7 Nephrology Center of Maryland, Baltimore, MD USA

**Keywords:** Social determinants of health, Dialysis, Emergency department, Non-adherence

## Abstract

**Background:**

Dialysis patients who miss treatments are twice as likely to visit emergency departments (EDs) compared to adherent patients; however, prospective studies assessing ED use after missed treatments are limited. This interdisciplinary pilot study aimed to identify social determinants of health (SDOH) associated with missing hemodialysis (HD) and presenting to the ED, and describe resource utilization associated with such visits.

**Methods:**

We conducted a prospective observational study with a convenience sample of patients presenting to the ED after missing HD (cases); patients at local dialysis centers identified as HD-compliant by their nephrologists served as matched controls. Patients were interviewed with validated instruments capturing associated risk factors, including SDOH. ED resource utilization by cases was determined by chart review. Chi-square tests and ANOVA were used to detect statistically significant group differences.

**Results:**

All cases visiting the ED had laboratory and radiographic studies; 40% needed physician-performed procedures. Mean ED length of stay (LOS) for cases was 17 h; 76% of patients were admitted with average LOS of 6 days. Comparing 25 cases and 24 controls, we found no difference in economic stability, educational attainment, health literacy, family support, or satisfaction with nephrology care. However, cases were more dependent on public transport for dialysis (*p* = 0.03). Despite comparable comorbidity burdens, cases were more likely to have impaired mobility, physical limitations, and higher severity of pain and depression. (*p* < 0.05).

**Conclusions:**

ED visits after missed HD resulted in elevated LOS and admission rates. Frequently-cited SDOH such as health literacy did not confer significant risk for missing HD. However, pain, physical limitations, and depression were higher among cases. Community-specific collaborations between EDs and dialysis centers would be valuable in identifying risk factors specific to missed HD and ED use, to develop strategies to improve treatment adherence and reduce unnecessary ED utilization.

## Background

Almost half of hospital-associated medical care in the United States is delivered in the Emergency Department (ED) [1]. Overall, EDs in the United States treat over 135 million patients annually [2]. With subsequent rising costs and ED overcrowding, there is an increased focus on identifying patients at higher risk of frequent, possibly preventable, visits [3–5]. Such patients include those undergoing dialysis for end-stage renal disease (ESRD) [3, 6]. Over the past several decades, the number of patients receiving dialysis for ESRD in the United States has increased significantly, from about 56,000 in 1980 to more than 500,000 in 2016 [7]. Consequently, the incidence of ED care for ESRD patients has also increased and is now six to eight times greater than that of the general population, with up to twice the ED length of stay and significantly higher admission rates [8–10]. Among ESRD patients who shorten or miss dialysis treatments, the risk of ED care further doubles and the risk of re-hospitalization quadruples; missed dialysis treatments are associated with all-cause mortality and worse health [11–14].

Despite growing ED utilization by dialysis patients and its attendant cost and morbidity, prospective studies to identify risk factors for ED utilization by dialysis patients who have missed dialysis treatments remain limited. Patients on scheduled maintenance dialysis miss treatments and present to the ED despite established weekly points of contact with the outpatient healthcare system. Access to ambulatory care does not fully explain the risk of ED visits in ambulatory care sensitive conditions; elements such as income, ethnicity, cultural background, and trust with the healthcare system impact ED utilization [15–18]. Similarly, community-level determinants may play a role in dialysis non-adherence and ED utilization [19]. The World Health Organization (WHO), Centers for Disease Control (CDC), and the Healthy People 2020 and 2030 initiatives have all placed an increased focus upon social determinants of health (SDOH), i.e. the conditions in the places where people live, learn, work, and play. SDOH impact patients’ behavioral choices and are associated with clinical outcomes in ESRD patients [20–23]. Unmet SDOH in the ESRD patient may predict non-adherence to dialysis treatment and the incidence of preventable ED visits or hospitalizations. However, SDOH have not received sufficient attention in ESRD patients, and there is very limited prospective data on SDOH and their impact on dialysis non-adherence and ED utilization [20]. The urban ED, which serves as a “window into the community” and a social safety net, is poised to address the aforementioned research gaps [24, 25]. Therefore, this pilot study seeks to identify the specific medical factors and SDOH associated with missing hemodialysis (HD) and subsequent ED utilization, and to describe the resultant resource use upon presenting to the ED.

## Methods

### Study design, setting, and population

This pilot interview-based study used a prospective observational design with a control group. Cases (patients who had missed at least one HD session prior to ED visit) were recruited in the ED of a large academic center (site 1). Controls (patients deemed adherent by their nephrologist) were recruited from a population of those regularly attending a large local outpatient HD clinic (site 2).

Both sites are located in the same large urban city and serve communities in the same urban setting. Site 1 is an ED at an academic, tertiary care center with approximately 70,000 visits per year and a 22% admission rate. Site 2 is a large outpatient HD center in the same city with nearly 300 chronic outpatient HD patients. Sessions are offered daily, with the exception of Sundays. The study was approved by the institutional review boards at both institutions.

Patients were eligible to participate as cases if they had missed at least one HD session prior to ED visit at site 1, were over age 18, and spoke English. Patients who had already participated, were deemed critically ill by ED clinicians, or were unable to consent were excluded.

After chart review and documentation review by their treating nephrologists, patients at site 2 who had missed 0 dialysis sessions in the year preceding recruitment were identified as potential controls. Controls were matched to cases to ensure a similar distribution of gender, age, diagnosis of diabetes as a comorbidity and years on dialysis. Controls were eligible for inclusion if they were aged over 18 and spoke English.

### Study protocol and outcome measures

Trained research staff at site 1 prospectively identified cases, i.e. patients who may have missed HD, by reviewing patient charts of current visits in the electronic medical record (EMR) and obtained the treating clinician’s permission prior to approaching patients. When research staff was not on-site, ED clinicians notified research staff of potentially eligible patients after obtaining patient permission to be contacted. Research staff then contacted potentially eligible patients either while in the ED, while admitted to the hospital, or over the phone after hospital discharge if the patient became too ill for interview completion during the ED visit or if the patient left the ED prior to interview completion. Consenting participants completed interviews either in-person or over the phone. Written consent was obtained from participants recruited in-person, and verbal consent using a standardized script was obtained from patients recruited over the phone. Both consent strategies were approved by both institutional review boards.

Research staff approached eligible controls at site 2 for recruitment during their scheduled regular outpatient HD sessions and participants providing written consent completed interviews in-person.

Both cases and controls completed the same interview with research staff, with the exception of additional questions for cases regarding reasons for missed HD and number of missed HD sessions prior to ED visit. Comorbidities, degree of disability, and depression were assessed using instruments validated in dialysis patients (the Charlson comorbidity index, Kidney Disease Quality of Life Instrument [KD-QOL], and Patient Health Questionnaire-9 [PHQ-9], respectively) [26–28]. Pain levels in preceding weeks were documented, along with alcohol and illicit substance use.

The interview subsequently gathered data on various categories of SDOH. Key domains aligned with the Healthy People 2020 Approach to SDOH categorizations were 1) economic stability, 2) neighborhood and built environment, 3) education, 4) healthcare access, and 5) social and community context [29]. Our measures of economic stability included employment status and the Distressed Communities Index (DCI) [30]. The DCI combines seven metrics (high school diploma, housing vacancy rate, unemployment, poverty rate, median income ratio, change in employment, change in business establishments) based upon zip-code to generate a measure of community economic well-being. Neighborhood and built environment were examined with the DCI, mode of transportation to HD, and distance to HD center. Education was assessed by highest level of formal education achieved. Health care access was assessed via health literacy with a brief questionnaire (Rapid Estimate of Adult Literacy in Medicine Short Form or REALM-SF), which has been validated in the dialysis population [31, 32]. Social and community contexts were captured by determining level of family support, availability of back-up plans for missed HD, and satisfaction with care (with the Choices for Healthy Outcomes in Caring for End-Stage Renal Disease Satisfaction Questionnaire) as a surrogate for attitude and trust towards the medical system [33, 34].

In both populations, EMR chart reviews were used to verify patient comorbidities. For cases, data on patient disposition, length of stay, and resource use were gathered from EMR chart review. Chart review was conducted by trained abstractors using a standardized abstraction form. A sample of charts (20% from each group) was reviewed by a study author (KB) to ensure accuracy.

### Data analysis

Data were collected and managed using REDCap electronic data capture tools hosted at Johns Hopkins University [35]. All data were checked for consistency and outliers were examined. Two sample tests of proportions and chi-square analysis or test of medians when appropriate were used to identify significant (*p* < 0.05) differences between cases and controls. STATA 12.0 (Stata Corp, College Station, TX) was used for analysis.

## Results

Thirty-two eligible cases were identified; four declined to participate and study team members were not able to reach three patients by phone after their hospital visit. Twenty-eight controls were identified; three declined to participate and one was hospitalized during the recruitment period for a non-hemodialysis related cause. In sum, 25 cases and 24 controls were successfully recruited and completed the study.

In our recruitment process, we controlled for gender, age, diabetic status, and years on dialysis, and, accordingly, groups did not differ significantly in these factors. (*p* > 0.05) (Table 1) Most participants were African-American and had been on HD for less than 5 years. 44.9% were female; 32.7% were diabetic.

**Table 1:** Baseline patient characteristics, comorbidities, mobility, pain, depression, and substance use

Characteristic	Cases	Controls	*P*-value
Female patients – n (%)	11 (44.0)	11 (45.8)	0.897
Age - mean years (SD)	53.68 (12.77)	55.42 (10.64)	0.7564
Hemodialysis vintage - n (%)			0.639
< 1 year	4 (16.0)	5 (20.8)	
1–5 years	15 (60.0)	10 (41.7)	
**>** 5–10 years	4 (16.0)	6 (25.0)	
> 10 years	2 (8.0)	3 (12.5)	
Ethnicity - n (%)			0.972
African-American	24 (96.0)	23 (95.8)	
Caucasian	1 (4.0)	1 (4.2)	
Comorbidities - n (%)			
Diabetes	8 (36.0)	8 (33.3)	0.844
Hypertension	18 (72.0)	24 (100.0)	0.008
Coronary artery disease	7 (28.0)	5 (20.1)	0.523
Congestive heart failure	7 (28.0)	8 (33.3)	0.690
Charlson Comorbidity Index - median (IQR)^b^	6 (4–8)	5 (3.5–7)	0.23
Degree of mobility - n (%)			
Fully mobile without assistance	8 (32)	20 (83)	< 0.001
Reliant on cane	11 (44)	3 (12.5)	0.015
Reliant on walker	8 (32)	1 (4.2)	0.012
Reliant on wheelchair	2 (8)	0 (0)	0.157
Health limitation score (KD-QOL) median (IQR)^a,b^	35 (10–85)	60 (45–95)	0.02
Degree of bodily pain in preceding weeks - n (%)			< 0.001
None	3 (12)	10 (41.7)	
Very mild/mild	3 (12)	11 (45.8)	
Moderate	3 (12)	1 (4.2)	
Severe/very severe	16 (64)	2 (8.3)	
PHQ-9 Depression score - n (%)			0.016
None	7 (28)	17 (70.8)	
Mild	7 (28)	5 (20.8)	
Moderate	4 (16)	2 (8.3)	
Moderately Severe	5 (20)	0 (0)	
Severe	2 (8)	0 (0)	
Alcohol and substance use			
Ever use illicit drugs	12 (48)	7 (29.2)	0.176
Ever use alcohol	14 (56)	7 (29.2)	0.06
In methadone/suboxone program	3 (12)	0 (0)	0.08
Currently use alcohol	1 (4)	2 (8.3)	0.527
Currently use illicit drugs	5 (20.8)	1 (4.2)	0.081

^a^lower scores signify greater limitations

^b^25–75% interquartile range

### Characteristics of ED visits by patients who missed HD

Among cases, the most common reasons for missing HD included feeling unwell or having issues with transportation. Notably, three out of seven patients reporting transportation issues had difficulties related to the state mobility program. (Table 2) Fourteen cases (56%) had missed one session of HD prior to ED presentation, whereas five (20%) had missed two sessions and six (24%) had missed three or more sessions. The most frequent complaint on presentation was shortness of breath (six patients, 24%) and six patients (24%) were acuity level 2 on the Emergency Severity Index (ESI) scale, whereas the remainder were level 3 (ESI level 1 represents highest acuity, with 5 being lowest) [36]. Most patients arrived by private vehicle (12, 48%) or ambulance (9, 36%).

**Table 2:** Reasons provided for missing dialysis

Reason for missing dialysis	n (%)
Not feeling well	6 (21)
Transportation issue	7 (24)
Bad weather	1 (4)
Social or family obligations	4 (16)
Systems issue (insurance, lack of chair at center, etc)	6 (24)
Vascular access problem	2 (8)
Physical limitations	3 (12)

While in the ED, all patients had laboratory studies drawn, received at least one radiographic study, and had at least one specialty consulting service involved in their care. 52% required intravenous medications, and 32% required intravenous access placed via ultrasound guidance by an emergency department physician. 76% of patients subsequently had an in-patient stay; only 24% of patients were directly discharged from the ED with a median length of stay of 14 h. (Table 3) Almost half of admitted patients were placed in monitored units (48%), while 16% required an intensive care unit admission at some point during their inpatient stay. Median inpatient length of stay was up to 6 days.

**Table 3:** Disposition of cases presenting to the emergency department; LOS = length of stay

Disposition	n (%)	Total LOS in hours - Median (25–75% IQR)
Discharged from ED	6 (24)	14 (12–22)
Observation status in ED	1 (4)	12.0
Inpatient		
Observation	4 (16)	29 (29–33.5)
Full admission as inpatient	14 (56)	142.5 (85–221)
If inpatient, type of unit^a^		
Non-monitored	5 (20)	144 (72–92)
Monitored	12 (48)	84 (60–156)
Intermediate care unit	1 (4)	72.0
Intensive care unit	4 (16)	48 (24–84)

^a^some patients spent time in more than one unit (level of care upgraded or transferred)

### Medical factors associated with ED visits after missing dialysis

No significant differences were noted between groups in comorbidity burden as assessed by the Charlson Comorbidity Index (*p* = 0.23). (Table 1) However, cases were significantly less likely to be fully mobile (*p* < 0.001), had greater reliance on mobility adjuncts (*p* = 0.015, 0.012), and had poorer scores on the healthcare limitations scale as measured by the KD-QOL (*p* = 0.02). Cases also had significantly higher levels of pain, with the majority (64%) expressing severe or very severe bodily pain in the preceding 4 weeks (*p* < 0.001). Most controls were scored as having no depression on PHQ-9 screening, whereas cases had significantly higher rates of moderate (16%), moderately severe (20%), or severe depression (8%) (*p* = 0.016). No significant differences in patterns of alcohol or drug use were found between groups (*p* > 0.05); however, current participation in methadone or suboxone programs among cases trended towards significance (*p* = 0.08) (Table 1).

### Social determinants of health associated with ED visits after missing dialysis

Groups did not differ significantly in economic stability as measured by employment status and the Distressed Communities Index (DCI) (*p* = 0.749). (Table 4) Most patients were receiving disability or retired, with no significant difference in distribution between groups (*p* = 0.418). 44.9% of all subjects lived in distressed communities, with another 36.7% living in at-risk communities. There was no difference noted in overall distribution between groups. Cases had a larger proportion living in distressed communities compared to a larger proportion of controls in the at-risk tier, but this difference was not statistically significant. Moreover, these tiers are adjacent in ranking in the DCI.

**Table 4:** Economic stability and neighborhood, education, and healthcare access

Characteristic	Cases	Controls	*P*-value
Current employment status - n (%)			0.418
Full-time	0 (0.0)	2 (8.3)	
Part-time	1 (4.0)	2 (8.3)	
Unemployed	2 (8.0)	2 (8.3)	
On-disability	15 (60.0)	15 (62.5)	
Retired	7 (28.0)	3 (12.5)	
Distressed communities index - n (%)			0.749
Prosperous	2 (8.0)	1 (4.2)	
Comfortable	2 (8.0)	2 (8.3)	
Mid-tier	1 (4.0)	1 (4.2)	
At-risk	7 (26.9)	11 (45.8)	
Distressed	13 (52)	9 (37.5)	
Mode of transport to dialysis - n (%)			
Drive self	4 (16)	14 (58.3)	0.002
Public transportation	7 (28)	1 (4.2)	0.024
State mobility service	9 (36)	3 (12.5)	0.056
Walk	2 (8)	1 (4.2)	0.576
Rides from family/friends	6 (24)	1 (4.2)	0.047
Other	2 (8)	4 (16.7)	0.355
Distance from dialysis center - n (%)			0.09
1 mile or less	4 (17.4)	6 (25.0)	
1–5 miles	10 (43.5)	15 (62.5)	
> 5 miles	9 (39.1)	3 (12.5)	
Unsure	2 (8.0)	0 (0)	
Educational attainment - n (%)			0.872
Some high school	7 (28.0)	5 (20.8)	
Completed high school/obtained GED	7 (28.0)	7 (29.2)	
Some college	7 (28.0)	9 (37.5)	
College degree or postgraduate degree	4 (16.0)	3 (12.5)	
Health literacy (REALM-SF) - n (%)			0.831
3rd grade and below	1 (5.6)	2 (8.3)	
4th–6th grade	3 (16.7)	3 (12.5)	
7th–8th grade	4 (22.2)	8 (33.3)	
High school	10 (55.6)	11 (45.8)	

Controls were significantly more likely to drive themselves when going to HD (*p* = 0.002), whereas cases were more likely to rely on public transportation (*p* = 0.024). However, there was no significant difference between groups in distance traveled to their outpatient HD center from home (*p* = 0.09). We did note a larger proportion of cases living more than 5 miles from their outpatient HD center, but this difference was not statistically significant. Our sample size did not permit differentiation by mode of transport when examining impact of distance.

There was no significant difference in maximum educational attainment between groups (*p* = 0.872), and groups did not differ in degree of health literacy as measured by REALM-SF (*p* = 0.831). (Table 4) Health literacy was assessed for a convenience sample of cases, since six interviews were conducted over the phone. Respondents were also asked about patterns of ED use. Cases were more likely than controls to report visiting the ED multiple times a year for any medical problem. (*p* = 0.02).

The majority of respondents (70.8% of controls and 64.0% of cases) reported that their family was very involved in their medical care, and the majority reported that their families were somewhat or very supportive of their medical care (95.9% of controls and 92% of cases), with no significant difference between groups. Most patients reported having a reliable back-up plan if unable to get to HD, with no significant difference between groups (63.2% of cases and 62.5% of controls). Overall, groups did not differ significantly in how frequently they assigned an “excellent” rating to their nephrologists, outpatient HD center staff, and HD center as a whole. 91.7% of cases and 75.1% of controls would probably or definitely recommend their dialysis center to others. (*p* = 0.099).

## Discussion

This prospective pilot study examines dialysis non-adherence and subsequent ED utilization through the lens of social determinants of health (SDOH). Non-adherent patients presenting to the ED had significantly higher levels of pain, depression, and limitations in mobility, despite comparable comorbidity burdens, and were more likely to rely on public transport, with economic stability and built environment similar to that of controls. Our findings suggest heavy ED resource use by such patients with higher-than-average admission rates, and likely underestimate resource consumption, as critically ill non-adherent patients were not included.

To date, there are very limited studies which prospectively identify risk factors predicting ED visits among patients who miss dialysis treatments. Existing literature is primarily retrospective, regional registry-based, and focused on all-comers, without specific consideration of the non-adherent dialysis patient [8, 9, 19, 37]. The limited number of studies examining HD non-adherence have been post-hoc analyses and have not specifically identified populations at risk of ED utilization after non-adherence [11, 38]. There also remains a paucity of studies examining the relationship of SDOH with dialysis adherence or directly engaging on patients on reasons for missed treatment sessions. Retrospective studies are limited in their ability to capture key SDOH, such as attitudes towards medical care, degree of community engagement, or social support. Furthermore, registry-based studies lack the granularity necessary to examining relevant SDOH within local contexts.

To address the aforementioned evidence gaps, we prospectively examined risk factors predicting ED visits among patients who had been non-adherent to dialysis. Despite a comparable comorbidity burden, non-adherent patients in our study had higher rates of physical limitations and limited mobility. Limitations in physical activity may influence health-related quality of life and independence, which could, in turn, be related to self-efficacy [39]. Self-efficacy has been associated with greater self-care and fewer missed dialysis appointments amongst ESRD patients and may mediate the impact of depression on adherence in other patient populations [40–43]. We noted higher rates of depression and pain among non-adherent dialysis patients. ESRD patients with co-morbid depression or pain are at higher risk of ED use, and pain has been cited as a potential risk factor for withdrawing from dialysis treatment [11, 38, 44]. However, depression may be under-recognized and under-treated in ESRD patients [45]. Dialysis patients should be longitudinally screened for both depression and pain, and managed appropriately.

As a measure of neighborhood and built environment, transportation is often cited as a powerful predictor for non-adherence. Accordingly, we found that non-adherent dialysis patients were far more likely to rely on public transportation, less likely to drive, and frequently reported that transportation issues led to missed HD treatments. Transportation barriers could be potentiated by the increased prevalence of pain and physical limitations among non-adherent patients. Although distance to dialysis may have impacted transport modality and adherence to treatment, we did not find a significant correlation. Prior studies have indicated an increased risk for missed treatments among patients using a transportation van or with longer drives to HD [11]. However, since we did not collect information regarding transit times, it was not clear if shorter transit times to appointments predicted adherence.

Education and economic stability (e.g., employment status, DCI) were not significantly associated with dialysis adherence. The latter is likely due to the fact that very few patients in both the adherent and the non-adherent groups were employed full-time. The majority of participants were receiving disability benefits or were retired. Nevertheless, 24% of cases reported that a systems issue related to healthcare access led to missed HD treatment. Further studies are needed to better understand the role of economic stability with dialysis adherence.

Although multi-site studies have shown a correlation between low health literacy and non-adherence, we did not detect a significant association [32, 38]. Similar focused, smaller studies have also failed to show a significant correlation between health literacy and preventable hospitalizations or ED visits [46]; as such, it is unclear whether health literacy itself impacts adherence, whether it serves as a surrogate for other SDOH, or whether these variations are attributed to context-specific factors. Furthermore, since most studies examining risk factors for non-adherence have been conducted across multiple study sites, the aggregation of the data may have resulted in the loss of local context and variations in SDOH. Similarly, we did not detect a significant association between satisfaction with care and non-adherence to dialysis. This suggests that trust towards the healthcare establishment is highly variable between communities, emphasizing the importance of local community context when examining the influence of SDOH.

### Limitations

Similar to other patient-centric pilot studies, we recognize that our primary limitation to generalization is our sample size. Despite the small sample size, however, our study offers a rich cross-section of data and provides a “portrait” of the non-adherent dialysis patient in our inner city major metropolitan setting, which may inform future studies and interventions. Our study population was predominantly African-American; while this may render our results applicable to similar metropolitan settings, they are not necessarily applicable to all settings or the US dialysis patient population at large. Our study did not attempt to capture all possible risk factors, such as tobacco use or marital status, but examined a representative sample of SDOH. Certain variables which trended towards but did not achieve statistical significance, such as distance to dialysis, may impact adherence. Moreover, our small sample size prevented the analysis of any possible associations between SDOH and reasons given by patients for missing sessions. Additionally, since critically ill patients were excluded from our study, it is possible that their HD non-adherence was the result of a significant medical illness, and not necessarily related to social determinants. Respondents may have been subject to recall and self-selection bias.

## Conclusions

Non-adherent dialysis patients who present to the ED require prolonged inpatient visits and use multiple resources while in the ED. The interactions and impacts of specific SDOH may vary by context, and further studies aimed at risk factor identification or intervention design should be locally-focused. This pilot study demonstrates the interdisciplinary collaborative potential between long-term care providers (nephrologists) and acute care providers (emergency medicine providers). This “across the continuum” approach may be key in identifying the most socially-vulnerable patients, evaluating the prevalence of unmet SDOH in such populations, optimizing adherence to treatment regimens, and influencing health-seeking behavior by designing comprehensive, context-specific interventions that can be conducted either in the outpatient setting or at point-of-care in the ED itself. Our findings suggest that an inter-professional approach, incorporating psychiatric services, social work, case management and pain management, may be most effective in addressing the complex, interrelated SDOH that contribute to these patterns of healthcare utilization.

## Data Availability

The datasets used during the current study are available from the corresponding author on reasonable request.

## Abbreviations

CDC: Centers for Disease Control
DCI: Distressed Communities Index
ED: Emergency department
EMR: Electronic medical record
ESRD: End-stage renal disease
HD: Hemodialysis
KQ-QOL: Kidney Disease Quality of Life
PHQ-9: Patient Health Questionnaire-9
REALM-S: Rapid Estimate of Adult Literacy in Medicine Short Form
SDOH: Social determinants of health
WHO: World Health Organization
